# Anticancer Effects of Lingonberry and Bilberry on Digestive Tract Cancers

**DOI:** 10.3390/antiox10060850

**Published:** 2021-05-26

**Authors:** Tuulia Onali, Anne Kivimäki, Matti Mauramo, Tuula Salo, Riitta Korpela

**Affiliations:** 1 Department of Oral and Maxillofacial Diseases, Faculty of Medicine, University of Helsinki, 00014 Helsinki, Finland; tuulia.onali@helsinki.fi (T.O.); anne.kivimaki@helsinki.fi (A.K.); tuula.salo@helsinki.fi (T.S.); 2 Medical Nutrition Physiology, Department of Pharmacology, Faculty of Medicine, University of Helsinki, 00014 Helsinki, Finland; 3 Translational Immunology Research Program (TRIMM), University of Helsinki, 00014 Helsinki, Finland; 4 Department of Pathology, Faculty of Medicine, University of Helsinki and Helsinki University Hospital, 00014 Helsinki, Finland; matti.mauramo@helsinki.fi; 5 Cancer and Translational Medicine Research Unit, University of Oulu, 90014 Oulu, Finland; 6 Medical Research Centre, Oulu University Hospital, 90014 Oulu, Finland; 7 Human Microbiome Research Program, University of Helsinki, 00014 Helsinki, Finland

**Keywords:** lingonberry, bilberry, colorectal cancer, oral cancer, proliferation, invasion, migration, tumorigenesis, phytochemical, polyphenol, anthocyanin, proanthocyanidin

## Abstract

Wild berries are part of traditional Nordic diets and are a rich source of phytochemicals, such as polyphenols. Various berry treatments have shown to interfere with cancer progression in vitro and in vivo. Here, we systematically reviewed the anticancer effects of two Nordic wild berries of the *Vaccinium* genus, lingonberry (*Vaccinium vitis-idaea*) and bilberry (*Vaccinium myrtillus*), on digestive tract cancers. The review was conducted according to the PRISMA 2020 guidelines. Searches included four databases: PubMed, Scopus, Web of Science, and CAB abstracts. Publications not written in English, case-reports, reviews, and conference abstracts were excluded. Moreover, studies with only indirect markers of cancer risk or studies with single compounds not derived from lingonberry or bilberry were not included. Meta-analysis was not performed. The majority (21/26) of studies investigated bilberry and colorectal cancer. Experimental studies on colorectal cancer indicated that bilberry inhibited intestinal tumor formation and cancer cell growth. One uncontrolled pilot human study supported the inhibitory potential of bilberry on colorectal cancer cell proliferation. Data from all 10 lingonberry studies suggests potent inhibition of cancer cell growth and tumor formation. In conclusion, in vitro and animal models support the antiproliferative and antitumor effects of various bilberry and lingonberry preparations on digestive tract cancers.

## 1. Introduction

Diets rich in non-starchy vegetables and fruits reduce the risk of many non-communicable diseases, such as digestive tract cancers [1,2]. Plant-based diets provide vitamins, minerals, and dietary fiber, but also various plant metabolites generally called phytochemicals, such as polyphenols and phenolic acids. These are food constituents not regarded as essential but recently shown to have health benefits, especially in the prevention and management of chronic diseases, including cancer [3,4]. Dietary plants contain thousands of different phytochemicals, which produce a variety of metabolites in the human digestive system [4]. Polyphenols can modulate carcinogenic processes, such as cellular proliferation, differentiation, apoptosis, inflammation, angiogenesis, and metastasis [3,5]. The anthocyanins especially raise interest as promising candidates for cancer prevention and drug development, yet evidence from human trials is still lacking [6].

Bioavailability of dietary polyphenols and other phytochemicals has been considered low, since only small concentrations can generally be found in systemic circulation following ingestion [4]. However, since dietary phytochemicals are extensively modified by host and microbial enzymes [4,7], post-prandial serum concentration of a certain phytochemical is not directly indicative of its biological impact, especially on the digestive tract tissues. Some phytochemicals are absorbed intact or as degradation products from the upper digestive tract, whereas others travel to lower digestive tract and produce absorbable metabolites hours later [4,7]. Absorbed compounds are further metabolized in the epithelial cells of the gastrointestinal wall and liver, after which these metabolites can continue to systemic circulation or return to the gut lumen due to enterohepatic circulation. Post-prandial serum concentration of a degradation product can be hundred-fold compared to that of the original phytochemical ingested [8]. In addition, phenolic compounds can accumulate into tissues over time [9,10].

Whole berry extracts and berry polyphenols purified from extracts, reduce tumor cell proliferation in vitro, and reduce tumor formation and growth in murine models [11]. Berry polyphenols not only exerts antioxidant potential but also inhibits inflammation, invasion, mutagenesis, and induces apoptosis in malignant cells. Some polyphenols inhibit glucose transport [12] and may therefore inhibit vigorously proliferating tumor cells that rely on high influx and metabolism of glucose [13].

Lingonberry (*Vaccinium vitis-idaea*) and bilberry (*Vaccinium myrtillus*) are common dietary fruits and one of the richest dietary sources of polyphenols, especially anthocyanins, in Finnish diets [14,15]. These berries belong to the genus *Vaccinium* but differ in their biochemical composition, with the proportion of anthocyanins being considerably high in bilberry and that of proanthocyanidins in lingonberry [16]. The objective of this systematic review was to collect current English-written scientific research on the anticancer effects of lingonberry and bilberry on digestive tract cancers. These effects of berries on digestive tract cancers have been extensively reviewed by Bishayee et al. [11], but to our knowledge, no systematic review encompassing anticancer effects of lingonberry or bilberry has been published to date.

## 2. Materials and Methods

Protocol of this review was registered and is available in PROSPERO (PROSPERO 2020CRD42020181924). Eligible studies included original research papers with the following components: 1. Lingonberry or bilberry treatment (fermented or not fermented whole berry or berry juice, whole berry extracts, anthocyanin-rich extracts, and other fractions of extracts derived from bilberry or lingonberry); 2. digestive tract human cancer cell line, animal model, or human patients; 3. treatment tested on a direct cancer-relevant endpoint, including the number and size of tumors or pre-cancerous lesions, inhibition of tumor cell proliferation, cytotoxicity, induction of apoptosis, or invasion and migration of tumor cells.

Publications not written in English, case-reports, reviews, and conference abstracts were excluded. Studies with only indirect markers of cancer risk, such as oxidative stress or inflammation, or studies with single compounds not derived from lingonberry or bilberry, were considered outside the scope of this review.

The literature search was carried out by 2 independent reviewers (TO and AK) on 20.4.2020 and updated on 4.3.2021 in four databases: PubMed, Scopus, Web of Science, and CAB abstracts. Search syntax for PubMed, Web of Science, and CAB was as follows: ((lingonberr* OR Vaccinium vitis-idaea OR cowberr* OR mountain cranberr* OR bilberr* OR vaccinium myrtillus OR whortleberr* OR european blueberr*) AND (neoplas* OR metasta* OR invas* OR migrati* OR malignan* OR polyp* OR aberrant crypt foci OR adeno* OR tumor* OR onco* OR cancer* OR carcino* OR *cancer OR mutage*)) In Scopus search was divided into two searches; bilberry: TITLE-Abs KEY ((bilberr* OR myrtillus OR whortleberr*) AND (neoplas* OR metasta* OR invas* OR migrati* OR malignan* OR polyp* OR “aberrant crypt foci” OR adeno* OR tumor* OR onco* OR cancer* OR carcino* OR *cancer OR mutage*)) and lingonberry: TITLE-ABS-KEY ((lingonberr* OR vitis-idaea OR cowberr* OR “mountain cranberr*”) AND (neoplas* OR metasta* OR invas* OR migrati* OR malignan* OR polyp* OR “aberrant crypt foci” OR adeno* OR tumor* OR onco* OR cancer* OR carcino* OR *cancer OR mutage*)).

Duplicates were removed and titles and abstracts were screened by two independent reviewers (TO and AK). Full texts were retrieved for included studies and for papers that did not provide all relevant information in the abstract. A final selection was made in duplicate based on the full text.

Data from each included study was extracted by 2 independent reviewers (TO and AK/MM) using a piloted form. Each study was extracted in duplicate. Disagreements were solved with the 3rd reviewer (AK/MM). Data collected from each study included publication information, authors, cell line/animal model/human population and site of cancer, berry species, berry preparation used for exposure, time of exposure, concentration, end points studied, methods used, controls, number of repeats and replicates (cell studies) or number of studied individuals (animals or human subjects), results, conclusions made by authors, reviewers’ evaluation of study quality, and other comments. Due to variable methodology across studies, collected data was considered suitable for qualitative synthesis only. Data was tabulated by cancer type (colorectal or oral cancer) and study type (human study, animal study, or in vitro study).

## 3. Results

**Figure 1:**
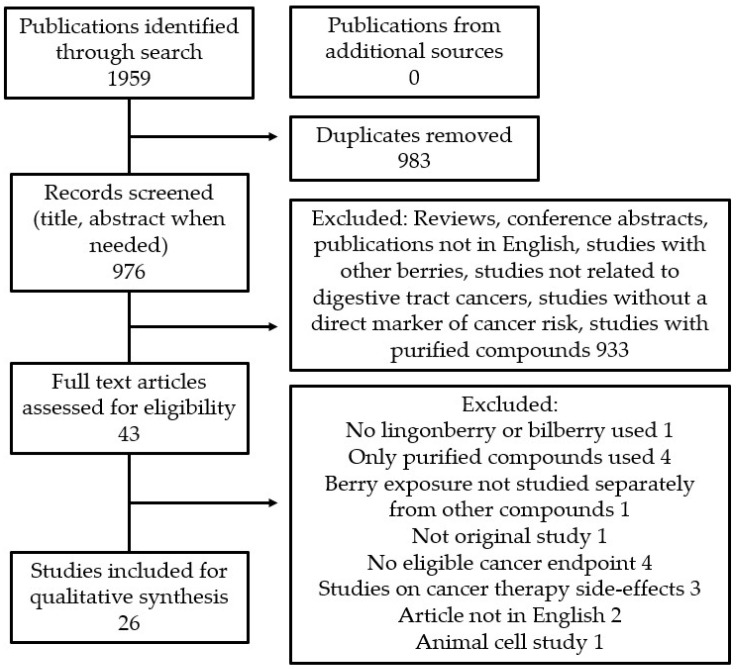
Flow chart of the systematic search and selection process.

The selection process of the systematic search and numbers of included and excluded papers are presented in Figure 1. In total, 43 full text articles were assessed for eligibility, of which 17 [17,18,19,20,21,22,23,24,25,26,27,28,29,30,31,32,33] were excluded based on reasons given in Figure 1. At the end of the selection process, 26 articles were considered eligible for this review. Of these studies, a great majority (25/26) investigated colorectal cancer (CRC); only one cell culture study related to oral cancer was identified, and no publications on other digestive tract cancers with a lingonberry or bilberry treatment were found. Anticancer effects of bilberry preparations on CRC were studied in one human clinical study, 7 animal studies, and 13 cell culture studies. Lingonberry treatment was tested on CRC models in one animal study and 8 cell culture studies. Study characteristics and main results from animal studies related to CRC are presented in Table 1 and from cell culture studies in Table 2.

### 3.1. Human Study on Colorectal Cancer

To our knowledge, only one clinical pilot human study on bilberry or lingonberry and any digestive tract cancer has been published to date. Thomasset et al. treated CRC patients with standardized bilberry anthocyanin rich extract, Mirtocyan (Indena S.p.A., Milan, Italy), for seven days before scheduled resection of primary tumor or liver metastasis [10]. Patients were randomized into three groups based on dosage (1.4/2.8/5.6 g/day), but no control group was included. Cellular proliferation and apoptotic index in the tumor tissue were compared to pre-intervention values. After the one-week treatment, tumor cellular proliferation decreased by 7% and apoptotic index increased from 3.6% to 5.3% in all groups combined.

### 3.2. Animal Studies on Colorectal Cancer

Animal experiments on CRC are presented in Table 1. Animal models used were genetic Apc^Min/+^ mouse model [9,34,35], toxin-induced carcinogenesis in a rat or mouse [34,35], and a xenograft mouse model with subcutaneously inoculated human adenocarcinoma cells [36,37]. Treatments included freeze-dried lingonberry and bilberry [38], as well as anthocyanin-rich bilberry extracts [9,34,35,36,37,39]. All these treatments reduced number and/or size of intestinal tumors or pre-malignant lesions. However, bilberry extract alone did not have an effect on subcutaneous tumors in the mouse xenograft, but reduced tumor volume when given in combination with an immune checkpoint inhibitor drug [36,37].

**Table 1:** Colorectal cancer: Main results of animal studies.

Publication	Berry	Population/Model	Endpoint	Method	Treatment	Effect
Lala et al., 2006 [34] (a)	Bilberry	Fisher 344 male rats treated with azoxymethane *n* = 10 per group	1. Number and multiplicity of colonic aberrant crypt foci (ACF) 2. Colonic cell proliferation	1. Staining and light microscopy of colons 2. Proliferating cell nuclear antigen immunohistochemistry	AIN-93 powdered diet supplemented with 3.85 g monomeric anthocyanin bilberry ARE/kg for 14 weeks	1. Significantly reduced number of total and large ACF compared to control. Number of large ACF was reduced by 70% 2. Significantly decreased colonic cellular proliferation by nearly 50%
Cooke et al., 2006 [9] (b)	Bilberry	Apc^Min/+^ mice *n* = 16 per group	Number, location, and size of adenomas in gastrointestinal tract	Dissection after termination	Standard diet with ARE from bilberry (Mirtoselect): Group 1: 0.03%, Group 2: 0.1%, Group 3: 0.3% *w*/*w* of ARE in diet for 12 weeks	Significantly and dose-dependently reduced adenoma load compared to control (number reduced by 30% with highest dose). Reduced particularly the number of small adenomas dose-dependently in the small intestine
Misikangas et al., 2007 [38] (b)	Bilberry and lingonberry	C57BL/6J Min/+ mice, male and female *n* = 10–12 in group	Sum of adenoma areas	Dissection after termination	10% *w*/*w* freeze-dried bilberry or lingonberry in High-fat AIN93 diet for 10 weeks	Both berries significantly inhibited number of adenomas by 15–30% compared to control. Bilberry did not reduce the size of adenomas, but lingonberry reduced adenoma burden by 60%
Lippert et al., 2017 [35] (a)	Bilberry	Female Balb/c mice, Azoxymethane/dextran sodium sulphate mouse model *n* = 50 mice divided in 3 groups	Tumor growth and number	Colonoscopy at weeks 4 and 9, macroscopic and microscopic analysis after termination at week 9	Bilberry anthocyanin-rich extract 1% or 10% *w*/*w* of extract in diet for 10 weeks	Significantly smaller and less (almost no detectable tumors) in 10% ARE fed mice compared to controls or mice fed with 1% extract. Smaller and less tumors also with 1% extract compared to control, but difference not statistically significant
Mudd et al., 2020 [39] (b)	Bilberry	Apc^Min/+^ mice treated with antibiotics and infected with enterotoxigenic *Bacteroides fragilis* *n* = 6 per group	Tumor number	Dissection after termination	Bilberry anthocyanin extract on average 8.6 mg/kg body weight by gavage 3 times a week for 4 weeks	Significantly reduced tumor number approx. 50% compared to control
Wang et al., 2020 [37] (c)	Bilberry	Female C57BL/6 mice subcutaneously inoculated with MC38-OVA cells, receiving immune checkpoint inhibitor injections *n* = 6 per group	Tumor volume	Measured every 3–4 days with an electronic caliper	Bilberry anthocyanin extract 156 ug of anthocyanins daily for 27 days	Extract alone did not affect tumor volume compared to control, but significantly enhanced the effect of the drug, possibly through modulation of gut microbiota (effect was abolished by antibiotic treatment)
Liu et al., 2020 [36] (c)	Bilberry	Female C57BL/6 mice subcutaneously inoculated with MC38-OVA cells, receiving immune checkpoint inhibitor injections *n* = 6 per group	Tumor volume	Measured every 3–4 days with an electronic caliper	Standardized bilberry ARE (Mirtoselect, Indena S.p.A. Italy) 25 mg bilberry extract/kg body weight daily for 2 weeks	Extract was not tested alone but enhanced therapeutic effects of the drug. Enhanced tumor immune filtration was associated with improvement of tumor control.

Animal model type used: (a) induced carcinogenesis, (b) genetic model, and (c) xenograft.

In the rat model of toxin-induced carcinogenesis, bilberry anthocyanin rich extract supplementation significantly reduced the number of total and large colonic aberrant crypt foci (ACF) after 14 weeks of treatment [34]. Proliferation of colonic cells reduced by nearly 50% and rats fed with this extract had reduced expression of COX-2 mRNA, together with a reduced number of ACF. In a corresponding mouse model study, bilberry anthocyanin rich extract supplementation for 10 weeks significantly reduced the size and number of colon tumors [35]. Interestingly, animals fed with 10% of this extract had almost no detectable tumors at the end of the trial. Reduction in colon length was measured as an indicator of inflammation and was attenuated together with tumor formation and growth by bilberry treatment.

Bilberry anthocyanin rich extract was effective also in the genetic Apc^Min/+^ mouse model, reducing dose-dependently the number of small intestinal adenomas [9]. In another study using this mouse model, freeze dried bilberry reduced only the number, but interestingly not the size of adenomas [38]. Similarly, the number of tumors was reduced by bilberry anthocyanins in Apc^Min/+^ mice treated with antibiotics and subsequently infected with enterotoxigenic *Bacteroides fragilis*, but tumor size was not reported in this study [39]. Freeze dried lingonberry, however, reduced both the number and size of intestinal adenomas [38]. This was the only animal study conducted with a lingonberry treatment and the only study with a whole-berry preparation instead of an anthocyanin rich extract.

In the two studies on a mouse xenograft with subcutaneous CRC tumors, bilberry anthocyanin rich extract alone did not affect tumor volume but enhanced the effectivity of an immune checkpoint inhibitor drug in combination therapy [36,37]. Enhancement of tumor control was associated with tumor immune filtration in the study of Liu et al., and was abolished with antibiotic treatment in Wang et al. publication, suggesting a role for modulation of gut microbiota and/or immune responses by bilberry anthocyanin rich extract [36,37].

In these reviewed animal studies, formation and growth of intestinal tumors or pre-cancerous lesions was inhibited with 10% (*w/w*) of freeze-bilberry or lingonberry in diet [38], from a few grams/kg to 10% (*w*/*w*) of bilberry anthocyanin extract in diet [9,35], and with 3.85 g of bilberry anthocyanin rich extract/kg in diet [34]. When administered separately, 8.6 mg/kg of bilberry anthocyanin extract 3 times per week reduced tumor number by approximately 50% compared to the control [39]. In the studies on xenograft models, 156 µg/day per mouse and 25 mg/kg body weight daily of bilberry anthocyanin extract were sufficient for enhancing a therapeutic effect of an immune checkpoint inhibitor drug [36,37].

### 3.3. In Vitro Studies on Colorectal Cancer

Studies on CRC cell lines are presented in Table 2. A total of 10 studies with a bilberry treatment, 5 with a lingonberry treatment, and 3 with both were identified. In these studies, berry extracts were found to reduce CRC cell proliferation [39,40,41,42,43,44,45,46,47,48,49,50,51] and induce apoptosis [44,49,52]. Furthermore, tumor cells were more sensitive than non-tumorigenic cells to the anti-proliferative effects of investigated berry extracts [39,43].

**Table 2:** Colorectal cancer: Main results of in vitro studies.

Study	Berry	Cell Line	Endpoints	Methods	Exposure	Effect
Katsube et al., 2003 [41]	Bilberry, lingonberry	HCT116	1. Cell growth inhibition 2. Induction of apoptosis (for bilberry only)	1.Cell counting by trypan blue exclusion 2. DNA extraction and agarose gel electrophoresis/Analysis of nuclear morphology by 1 mM bisbenzimide staining	1. 2–4 mg dry weight/mL of bilberry/lingonberry ethanol extract for 48 h, in addition bilberry ethanol extract for 24 and 48 h with 0.5–4 mg dry weight/mL 2. 4 mg/mL bilberry extract for 24 h	1. Bilberry and lingonberry ethanol extracts strongly inhibited the growth of HCT116. Bilberry inhibited cell growth >50% with 2–4 mg/mL. Over 50% inhibition was achieved also with 4 mg/mL lingonberry extract 2. Bilberry extract did not induce apoptosis
Olsson et al., 2004 [42]	Lingonberry	HT-29	Cell growth inhibition	WST-1 assay	Lingonberry ethanol extract/anthocyanin fraction 0.025–0.5% of plant dry matter of total weight in the wells for 24 h	Decreased proliferation significantly and dose-dependently. GI50 between 0.25% and 0.5% of plant dry matter in the wells Anthocyanin fraction was less effective
Zhao et al., 2004 [43]	Bilberry	HT-29 NCNM460 (Immorta-lized colon cell line)	Cell growth inhibition	Sulphorhodamine B assay	Bilberry anthocyanin-rich extract (Artemis International Inc.) 25–75 µg of monomeric anthocyanin/mL for 24/48/72 h	Significantly and time-dependently inhibited growth of HT-29 with 50–75 µg/mL of monomeric anthocyanin from 24 h on, with 25 µg/mL from 48 h on. Over 50% inhibition for all concentrations after 72 h. NCNM460 was only inhibited after 72 h
Wu et al., 2007 [44]	Bilberry, lingonberry	HT-29	1. Cell growth inhibition 2.Induction of apoptosis	1.Total cell count determined using SYTOX-green 2.DNA fragmentation by agarose gel electrophoresis	1.5–60 mg/mL bilberry/lingonberry methanol extract for 24 h 2. 5–60 mg/mL bilberry extract for 48 h	1. Both inhibited cell growth significantly. Bilberry GI50 = approx. 15 mg/mL Lingonberry GI50 = 60 mg/mL 2. Bilberry induced apoptosis with 20–60 mg/mL
Jing et al., 2008 [45]	Bilberry	HT-29	Cell growth inhibition	Sulphorhodamine B assay	Bilberry anthocyanin-rich extract (Artemis International, Inc.) 0–200 µg/mL cyanidin-3-glucoside equivalents for 48 h	Dose-dependent inhibitory effect on the growth of HT29. GI50 = 32.2 µg/mL
McDougall et al., 2008 [46]	Lingonberry	Caco-2	Cell growth inhibition	Dojindo CCK-8 kit	Lingonberry acetonitrile extract (bound fraction from solid phase extraction only) 25–75 µg of gallic acid equivalents/mL for 72 h	Inhibited cell growth in a dose-dependent manner, GI50 38.3 µg GAE/mL
Schantz et al., 2010 [52]	Bilberry	HT-29 Caco-2	1.Cytotoxicity 2. Induced DNA damage	1. Alamar blue 2. Comet Assay	Bilberry extract from European bilberry pomace (Kaden Biochemicals, Hamburg, Germany) 1. 5–500 µg/mL for 1 and 24 h 2. 0.01–500 µg/mL for 1 and 24 h	1.Significant effect only on HT-29, with 500 µg/mL only. EC50 was not reached 2. Decreased induced DNA damage only in Caco-2 cells, with 5–100 µg/ml
Esselen et al., 2011 [47]	Bilberry	HT-29	1. Cell growth inhibition 2. DNA integrity 3. Drug interaction with topoisomerase poisons	1. Sulphorhodamine B assay 2. Comet assay 3. ICE assay with topoisomerase poisons Camptotecin and Doxorubicin	Bilberry industrial ARE (Indena, Milan, Italy) 1. 50–500 µg/mL for 72 h 2. 1–0 µg/mL for 1 h 3. 0.01–50 µg/mL, 30 min pre + 1 h coincubation	1. Bilberry ARE inhibited HT29 growth dose-dependently. GI50 was not reached 2. Bilberry ARE increased the rate of DNA strand brakes, but no additional oxidative damage observed 3.DNA-damaging effects and cytotoxicity of both drugs were inhibited by bilberry ARE
Fan et al., 2011 [48]	Lingonberry	HT-29	Cell growth inhibition	MTS assay	Lingonberry acetone extract 20–80 mg/mL for 48 h	Proliferation significantly inhibited in a dose-dependent manner, GI50 approx. 35 mg/mL
Kropat et al., 2013 [50]	Bilberry	HT-29	Cell growth inhibition	Living cells counted after staining with trypan blue	Pomace methanol extract from European bilberry 10–400 µg/mL for 72 h	Inhibited cell growth significantly, with concentrations above 100 µg/mL. GI50 between 200 and 400 µg/mL
Aaby et al., 2013 [49]	Bilberry	Caco-2 HT-29 HCT116	1. Cell growth inhibition/viability 2. Apoptosis (HT-29 only)	1. MTT Assay 2. Cell Death Detection ELISAPLUS Assay with BCA protein assay	Bilberry extract, raw juice and press residue extracts obtained from extraction in different temperatures (40/60/80/100 C) 1. 30 min exposure to 75–250 mg GAE/l. Measurement after 24 h 2. 24-h exposure with 75–250 mg GAE/l of press residue extracts	1. All extracts inhibited proliferation of all cell lines. Dose-response inhibition of all extracts on all cell lines, except for raw juice on HT-29 and HCT 116. GI50 of Caco-2 and HCT 116 was reached with all extracts, of HT-29 only press residue extracts from 80–100 °C temperatures 2. Dose response for both tested press residue extracts (extracted in 40/100 ºC temperature)
Brown et al., 2014 [53]	Lingonberry	HT-29 HT115	1. Cytotoxic activity 2. Inhibition of invasion and migration	1. MTT assay 2. Matrigel invasion and migration assays	Lingonberry methanol extract prepared to: IVDL: In vitro digested extract and IVFL: In vitro fermented extract both 3–50 µg/mL GAE for 24 h Ileal fluid after lingonberry ingestion, 3–200 µg/mL for 24 h	1. No cytotoxic activity for any exposure 2. IVDL and IVFL had significant anti-invasive effects, migration not affected
Šavikin et al., 2014 [54]	Bilberry	LS147	Cell viability	MTT assay	Bilberry decoction tea, bilberry infusion tea 12.5–200 µg/mL for 72 h	Both teas decreased viability Decoction tea EC50: 176.32 µg/mL Infusion tea EC50: 178.52 µg/mL
Tumbas Šaponjac et al., 2014 [51]	Bilberry	HT-29	Cell growth inhibition	Sulphorhodamine B assay	Bilberry extract fractions Fraction 1: Polar substances Fraction 2: 6 flavonols detected Fraction3: 8 phenolic acids identified 62.5–1000 µg of dried fraction/mL for 48 h	Fractions 2 and 3 suppressed cell growth significantly and dose-dependently. Over 50% growth inhibition achieved with ≥125 µg/mL of fraction 2, and with ≥500 µg/mL of fraction 3. Fraction 1 resulted in less than 10% inhibition with 250–500 µg/mL.
Minker et al., 2015 [55]	Bilberry, lingonberry	SW840 (primary) SW620 (metastasis)	Induction of apoptosis	Flow cytometry, cell surface phosphatidylserine detection, caspase 8 and caspase 9 activation	Bilberry/lingonberry proanthocyanidins extracted in acetone/methanol 5–75 µg/mL of each fraction during seeding and for 24 h for SW620 and 48 h for SW480	Bilberry induced apoptosis via extrinsic pathway EC50 for bilberry treated SW620: 24.7 µg/mL and SW480: 25.2 µg/mL EC50 for lingonberry treated SW620: 24.3 µg/mL SW480: 24.7 µg/mL
Borowiec et al., 2016 [56]	Bilberry	Caco-2	1. Cell viability 2. Genotoxicity	1. MTT assay 2. Single cell electrophoresis, also under oxidative stress (H2O2)	Bilberry juice extract (no solvent used) 1. 12.5–400 µg dry mass/mL for 48 h 2. 100 µg dry mass/mL 48 h	1. Viability of Caco-2 was significantly but modestly inhibited only with 400 µg/mL (approx. 20% inhibition) 2. Not genotoxic
Mudd et al., 2020 [39]	Bilberry	HCT 116 HT-29 CCD-18Co (Human colon)	Inhibition of proliferation/Cell viability	MTT assay	Bilberry anthocyanin extract concentrations up to 200 µmol/L	Bilberry anthocyanin extract inhibited proliferation of tumor cells more than colon cells IC50 values for HT-29: 124 µmol/L HCT-116: 75 µmol/L CCD-18Co: 1050 µmol/L
Vilkickyte et al., 2020 [40]	Lingonberry	HT-29	Cell viability	MTT assay	Lingonberry extracted in acetone and phenolic fractions subsequently isolated with column chromatography	Lingonberry extract fractions reduced viability with EC50 values approx. 0.05–1.1 mg/mL, Fraction 4 rich in proanthocyanidins being the most effective

The inhibition of cell proliferation was most commonly measured, and various bilberry extracts were effective in all studies in which their effect on the growth of CRC cells were analyzed [39,41,43,44,45,47,48,49,50,51]. Similarly, various lingonberry extracts inhibited proliferation in all studies which tested lingonberry extracts on CRC cell proliferation [40,41,42,44,46]. Within individual studies, higher concentrations of various berry extracts and longer exposure times generally led to higher inhibition of tumor cells, but similar consistency could not be seen when results of all studies were compared, possibly due to different chemical compositions of tested extracts. Only two studies compared inhibition of tumor cells to that of non-tumorigenic digestive tract cells, both indicating greater sensitivity of tumor cells [39,43]. Zhao et al. (2004) found that tumor cells responded to bilberry extract at lower concentrations (25µg of monomeric anthocyanin/mL) and at earlier timepoints (48 and 72 h) compared to non-tumorigenic cells, with significant growth inhibition of 24% and 53%, respectively [43]. Time-dependent inhibition was observed with higher concentrations (50 and 75 of monomeric anthocyanin/mL), whereas non-tumorigenic immortalized NCM460 cells were significantly inhibited only after 72 h with all concentrations. Similarly, in the study of Mudd et al. (2020), non-tumorigenic colon cells were less sensitive than tumor cells to bilberry anthocyanins, with IC50 values of 124 and 75 µmol/mL for tumorigenic HT-29 and HCT116, respectively, and 1050 µmol/mL for non-tumorigenic NCM460 cells [39].

Some in vitro studies reported cytotoxicity or cell viability instead of inhibition of proliferation, with inconsistent results [47,52,53,54,56]. Mild toxicity was reached with 400 ug/mL of bilberry juice on Caco-2 [56] and with 500 ug/mL of bilberry pomace extract on HT-29 CRC cells [52]. Bilberry decoction and infusion teas were cytotoxic to LS147 CRC cells with approximately 180 ug/mL EC50 values [54]. However, no toxicity was detected for 4 mg/mL of bilberry extract on HCT116 [41], or for in vitro digested or fermented lingonberry extracts on HT-29 or HT115 with concentrations below 50 ug/mL [53]. On the other hand, bilberry reduced toxicity of topoisomerase poisons in the study of Esselen et al., raising the possibility of counteracting therapeutic toxicity of chemotherapy [47].

Induction of apoptosis in CRC cells was also investigated [41,44,49,52]. Bilberry extracts induced apoptosis in HT-29 cells with concentrations above 20 mg/mL [44,49] but not in HCT116 with a concentration of 4 mg/mL [41]. Proanthocyanin-rich extracts of both lingonberry and bilberry induced apoptosis in SW820 and SW640 cells with much lower concentrations; EC50s for both extracts were below 25 ug/mL [55]. Apoptosis was further investigated in this study with bilberry proanthocyanidin extract and was found to be induced via the extrinsic pathway.

### 3.4. Oral Cancer

No eligible in vivo studies on lingonberry or bilberry and oral cancer were found, but one eligible study on oral carcinoma cells was identified (Table 3). Hoornstra et al. (2018) found that lingonberry juice fermented using *Saccharomyces cerevisiae* inhibited the proliferation of oral tongue cancer cell lines HSC-3 and SCC25 [57]. Fermented juice also inhibited the invasion of HSC-3, and similarly but not significantly that of SCC25. Migration, however, was not inhibited in either cell line.

## 4. Discussion

The aim of this work was to systematically review all in vitro and in vivo English written research publications related to the anticancer effects of lingonberry and bilberry on digestive tract cancers. Published data was mainly focused on bilberry and CRC, and these studies suggest antiproliferative and anti-tumor effect for various bilberry preparations. Only one uncontrolled pilot human trial was identified, in which bilberry anthocyanin-rich extract supplement modestly reduced cancer cells proliferation and increased apoptosis compared to pre-intervention values in CRC tissue after treatment as short as 7 days [10]. Lingonberry preparations showed inhibitory effects comparable to bilberry in cell culture models (Table 2), and freeze-dried lingonberry inhibited adenoma growth even more than bilberry in the only animal experiment conducted on lingonberry [38]. Fermented lingonberry juice inhibited the proliferation of oral carcinoma cells in vitro [57]. Taken together, evidence from human trials is still needed, but all data to date from in vitro and animal models support the antiproliferative and tumor-inhibitory effects of various bilberry and lingonberry preparations on digestive tract cancers.

In vitro, various bilberry extracts, even at low concentrations (GI50 values 25 µg/mL–5 mg/mL) inhibited proliferation [39,41,43,44,45,47,50,51], and at higher concentrations (above 20 mg/mL) induced apoptosis [41,44,49] in CRC cells. However, proanthocyanidin-rich extracts of bilberry and lingonberry induced apoptosis already at far lower concentrations, at the levels of micrograms/mL [55]. In vivo, the proliferation of CRC cells reduced by nearly 50 % in rats with induced carcinogenesis, treated with bilberry anthocyanin rich extract [34]. Small decrease in proliferation and increase in apoptotic index were also detected in tumor tissue of bilberry-treated CRC patients [10]. The specific mechanism behind the antiproliferative activity remains to be specified. Cell growth seems to be inhibited in a cytostatic manner in cell culture [41,43,44,57], but apoptosis may be induced after reaching a concentration threshold [44]. One animal study found that a reduction in colonic cell proliferation in bilberry-treated rats was associated with reduced expression of COX-2, suggesting a link with anti-inflammatory activity [34].

In the animal studies, bilberry extracts inhibited the formation of induced and genetic murine intestinal malignant tumors, or pre-malignant transformations [9,34,35,38,39]. Interestingly, bilberry did not reduce the size of small-intestinal adenomas in contrast to lingonberry [38]. In a later publication of this experiment, superior effect of cloudberry compared to bilberry on adenoma size was found to be associated with downregulated gene expression of energy metabolism-related genes and a smaller ratio of intraepithelial to all mucosal CD3+ T lymphocytes in the intestinal mucosa [33]. Bilberry did not affect the formation of subcutaneous tumors in a xenograft mouse model [36,37]. However, the bilberry extract enhanced the effectivity of an immune checkpoint inhibitor drug against subcutaneous tumors [36,37]. It is of note that tumors outside the digestive tract would likely be exposed to lower concentrations of berry phytochemicals and their metabolites than tumors in the digestive tract.

The studies we analyzed did not give an exact mechanistical explanation for antitumor activity of lingonberry or bilberry. In the one human pilot study, both cellular proliferation and apoptotic index in the tumor tissue were affected by bilberry extract supplementation [10]. According to the animal studies reviewed here, the anticancer effect of these berries is suggested to come across, at least in part, via modulation of gut microbiota and/or immune responses [33,35,36,37,39]. These effects could, to some extent, also impact tumorigenesis outside the digestive tract [36,37]. In addition to local effects, systemic effects could be reached via fluctuating postprandial pulses of phytochemicals, slow release of absorbable metabolites in the digestive tract, and accumulation to tissues during prolonged exposure. Besides inhibiting the growth of tumors, berry extracts may inhibit tumor initiation through their DNA-protecting abilities [11,53], supported by the lower number of tumors or pre-cancerous transformations in animals treated with lingonberry and bilberry extracts [9,34,35,38,39].

Anthocyanin-rich extracts are the most frequently examined berry extracts in digestive tract cancers. However, based on our reviewed articles, the anticancer effect of some other berry phytochemicals could be even stronger than that of anthocyanins [40,42,46], or the activity may be synergistic between different compounds. Interestingly, freeze-dried lingonberry reduced adenoma growth in the Apc^Min/+^ mouse model more than bilberry [38] regardless of the lower anthocyanin content in lingonberry. Additionally, both lingonberry and bilberry extracts inhibited CRC cell proliferation in vitro [41,44] regardless of their differing phytochemical profiles [16]. In fact, the anthocyanin-rich fraction of lingonberry extract was less antiproliferative than the whole lingonberry extract [32,33], or fractions rich in proanthocyanins [33,34]. In addition, bilberry flavonol fraction and phenolic acid fraction both inhibited proliferation of CRC cells, suggesting that antiproliferative activities are shared by different phenolic compounds [51].

The relevance of in vitro studies with berry extracts is debatable, since berry phytochemicals are extensively modified in the digestive tract, and preparations used in cell culture differ in their chemical composure from the metabolites formed during digestion. Therefore, in vitro studies with original berry extracts may have relevance in oral cavity cancers, but not necessarily in lower digestive tract neoplasia. Yet, based on effectivity shown in vivo (Table 1), and the potency of different extracts in vitro (Table 2 and Table 3), anticancer effects on digestive tract cancers may not require high systemic bioavailability of certain original berry phytochemicals. Some biological activity seems to persist even after extensive modification by human and microbial enzymes [53,57]; however, more research on this subject is needed.

The possible anticancer effects of lingonberry and bilberry are numerous, but exact mechanisms and effective constituents are still unclear and yet to be investigated in cancer prevention and treatment. Regardless of encouraging results from several in vitro and animal studies on bilberry and CRC, only one pilot human trial has been conducted. Deeper understanding of the effective lingonberry and bilberry components, their metabolites, synergy, and underlying biological mechanisms is needed to pave the way for human trials. Animal models have shown the potential of lingonberry and bilberry to inhibit CRC carcinogenesis, but effective dose for humans cannot be deduced based on animal studies. Proving preventive potential in human trials would require multicenter studies with large populations and long follow-ups. Elaborating the anticancer mechanisms of phytochemicals could reveal previously unseen or overlooked aspects of carcinogenesis, and help not only to improve cancer treatment, but also to assess and minimize the cancer risk of an individual. Possible therapeutic benefits of berry phytochemicals [36,37] and potential undesired interactions [47] in combination treatments should also be carefully investigated in future trials.

Due to varying methodology in reviewed studies, meta-analysis could not be conducted. Reporting of experimental replicates and repeats was unclear in some in vitro studies, and the statistical significance of the inhibition of cellular proliferation not always tested, but we did not exclude any controlled studies based on reporting quality. In contrast to our original inclusion criteria, one uncontrolled human pilot study was included, based on the lack of controlled human studies. It should also be noted that many berries have shown anticancer activities [11], but here we went through only research conducted on lingonberry and bilberry, not entirely covering the research field of anticancer effects of wild berries.

## 5. Conclusions

Based on the literature found, bilberry inhibits CRC cell proliferation and tumorigenesis in experimental models, but this effect has not yet been investigated in any placebo controlled human trials. Data also suggested similar activity for lingonberry on CRC and oral cancer cell lines. In addition to anthocyanins, various other berry phytochemicals may have similar and synergistic inhibitory effects on tumor processes. The identification of active berry components, exact local and systemic mechanisms of action, and effective doses require further investigation.

## Figures and Tables

**Table 3:** Oral cancer. Main results of the cell study.

Publication	Berry, Cell Lines	Endpoint	Treatment	Effect
Hoornstra et al., 2018 [57]	Lingonberry, HSC-3 SCC25	1. Cell proliferation with enzyme-linked immunosorbent assay, 5-bromo-2′-deoxyuridine BrdU Kit 2. Invasion with 3-D tumor myogel spheroid invasion assay	Lingonberry juice fermented using Saccharomyces Cerevisiae 500, 2500, 5000 ug/mL 48 h for proliferation 96 h for invasion	1. Significant inhibition at 2.5 and 5.0 mg/mL for both cell lines. GI50 1162 µg/mL for HSC-3, GI50 773 µg/mL for SCC-25 2. Significantly reduced invasion of HSC-3, invasion of SCC-25 was similarly inhibited but result was not significant

## Data Availability

No new data were created or analyzed in this study. Data sharing is not applicable to this article.
